# System Transparency in Shared Autonomy: A Mini Review

**DOI:** 10.3389/fnbot.2018.00083

**Published:** 2018-11-30

**Authors:** Victoria Alonso, Paloma de la Puente

**Affiliations:** ^1^ETSI Aeronáutica y del Espacio, Universidad Politécnica de Madrid, Madrid, Spain; ^2^ETSI Industriales, Universidad Politécnica de Madrid, Madrid, Spain; ^3^Centre for Automation and Robotics, Universidad Politécnica de Madrid-CSIC, Madrid, Spain

**Keywords:** transparency, shared autonomy, human-robot interaction, communication, observability, predictability, interface, user-centered design

## Abstract

What does transparency mean in a shared autonomy framework? Different ways of understanding system transparency in human-robot interaction can be found in the state of the art. In one of the most common interpretations of the term, transparency is the observability and predictability of the system behavior, the understanding of what the system is doing, why, and what it will do next. Since the main methods to improve this kind of transparency are based on interface design and training, transparency is usually considered a property of such interfaces, while natural language explanations are a popular way to achieve transparent interfaces. Mechanical transparency is the robot capacity to follow human movements without human-perceptible resistive forces. Transparency improves system performance, helping reduce human errors, and builds trust in the system. One of the principles of user-centered design is to keep the user aware of the state of the system: a transparent design is a user-centered design. This article presents a review of the definitions and methods to improve transparency for applications with different interaction requirements and autonomy degrees, in order to clarify the role of transparency in shared autonomy, as well as to identify research gaps and potential future developments.

## 1. Introduction

Shared autonomy adds to the fully autonomous behavior some level of human interaction, combining the strengths of humans and automation (Hertkorn, 2015; Schilling et al., 2016; Ezeh et al., 2017; Nikolaidis et al., 2017). In shared autonomy, humans and robots have to collaborate. Transparency supports a flexible and efficient collaboration and plays a role of utmost importance regarding the system overall performance.

In the next sections, current research about transparency in the shared autonomy framework is reviewed. The goal is to provide, by analyzing the literature, a general view for a deeper understanding of transparency which helps motivate and inspire future developments. The key aspects and most relevant previous findings will be highlighted.

Different ways of understanding transparency in human-robot interaction in the shared autonomy framework can be found in the state of the art. In one of the most common interpretations of the term, transparency is the observability and predictability of the system behavior, the understanding of what the system is doing, why, and what it will do next.

In section 2 the effect of levels of autonomy on transparency is analyzed. Then, the mini-review is organized according to the different ways of understanding transparency in human-robot interaction in the shared autonomy framework.

In section 3 transparency as observability and predictability of the system behavior is studied. Since the main methods to improve transparency are based on interface design and training, transparency is usually considered a property of such interfaces, and section 4 focuses on transparency as a property of the interface. Since natural language explanations are a popular way to achieve transparent interfaces, transparency as explainability is studied in section 5. Section 6 is dedicated to mechanical transparency, and ethically aligned design aspects of transparency are reviewed in section 7.

Hence, the wider and most extended interpretations and results are presented first, while more specific trends are left for later sections. This way, the reader can naturally focus on the general concepts before other implications are analyzed. A table of selected references for each section can be found at the end of the paper (Table 1).

**Table 1:** Summary.

**Transparency in shared autonomy**			
**2. Transparency and levels of autonomy**			
High autonomy		Low autonomy	Miller, 2014, 2017
Situation awareness	Low	High	Schilling et al., 2016; Ezeh et al., 2017
Transparency	Low	High	Kaber, 2017; Wright et al., 2017
Cognitive engagement	Low	High	Ososky et al., 2014; Yang et al., 2017
Risk of overtrust	High	Low	Desai, 2012; Chen et al., 2018
**3. Transparency as observability and predictability of system behavior**			
Transparency as observability of the system behavior.			Endsley, 2012, 2017
User understanding of what the system is doing, why, and what it will do next. User-centered design.			Kruijff et al., 2014; Hellström and Bensch, 2018; Villani et al., 2018
Transparency as the opposite of unpredictability			Miller, 2014; Iden, 2017
The robot's abilities, intent, and situational constraints are understood by the users. Legibility, Readibility			Takayama et al., 2011; Dragan et al., 2013; Busch et al., 2017; Wortham et al., 2017
Mechanism to expose decision making			Theodorou et al., 2017
Methods to establish Shared Situational Awareness and Shared Intentions (Interface Design and Training) Robot-to-Human and Robot-of-Human Transparency			Lyons, 2013; Lyons and Havig, 2014; Tsiourti and Weiss, 2014; Lorenz, 2015; Dragan, 2017; Wang et al., 2017; Doellinger et al., 2018; Javdani et al., 2018
**4. Transparency as a property of the Interface**			
Situation Awareness Transparency (SAT) Model.			Chen et al., 2014; Endsley, 2017
*Levels 1,2,3*: Perception, comprehension, and projection		
Multimodal interfaces			Perzanowski et al., 2001; Lakhmani et al., 2016; Oviatt et al., 2017
**Transparency as explainability**		
Transparency is the robot offering explanations of its actions. Route/planning/navigation verbalization			Kim and Hinds, 2006; Caminada et al., 2014; DARPA, 2016; Rosenthal et al., 2016; Wortham and Rogers, 2017
**5. Mechanical transparency**			
Wearable robots,exoskeletons,rehabilitation: Capacity to follow human movements without human-perceptible resistive forces Training, Avoiding over-trust			Robertson et al., 2007; Jarrasse et al., 2008; Jarrassé et al., 2009; van Dijk et al., 2013; Zhang et al., 2016; Awad et al., 2017; Beckerle et al., 2017; Bai et al., 2018; Borenstein et al., 2018; Fani et al., 2018
Telerobotics Realistic (transparent) perception of the remote environment.			Raju et al., 1989; Lawrence, 1993; Yokokohji and Yoshikawa, 1994; Ferre et al., 2007; Hirche and Buss, 2007; Slawinski et al., 2012; Goodrich et al., 2013; Hertkorn, 2015; Muelling et al., 2017
**6. Transparency and ethically aligned design**			
The ethical black-box			Winfield and Jirotka, 2017
Transparency as traceability and verification			Wortham et al., 2017
IEEE Global Initiative for Ethically Aligned Design			Bryson and Winfield, 2017
P7001-Transparency in Autonomous Systems			Grinbaum et al., 2017

## 2. Transparency and levels of autonomy

Traditionally, human-robot interaction in shared autonomy has been characterized by levels of autonomy (Beer et al., 2014). Sheridan and Verplank (1978) proposed an early scale of levels of autonomy to provide a vocabulary for the state of interaction during the National Aeronautics and Space Administration (NASA) missions. In later work, Endsley and Kaber (1999) and Parasuraman et al. (2000) established other levels of autonomy taxonomies, considering the distribution of tasks between the human and the system regarding information acquisition, decision making, and actions implementation.

Recently, Kaber (2017) reopened the discussion about whether levels of autonomy are really useful. This paper has received answers from Miller (2017) and Endsley (2018). Miller (2017) considers that levels of autonomy are only an attempt to reduce the dimension of the multidimensional space of human-robot interaction. Other authors agree with this multidimensional perspective (Bradshaw et al., 2013; Gransche et al., 2014; Schilling et al., 2016) and with the need of focusing on human-robot interaction (DoD, 2012). Endsley's reply (Endsley, 2018) is a review about the benefits of levels of autonomy.

For high levels of autonomy, when the system is operating without significant human intervention, additional uncertainty is expected. For high levels of autonomy, the user may have a low level of observability of the system behavior, and low predictability of the state of the system. The system might have a low level of transparency.

For low levels of autonomy, when the human operators are doing almost everything directly themselves, they know how the tasks are being carried out, so the uncertainty and unpredictability are typically low. Yet, the human operator's cognitive workload to be aware of everything increases to process all the information. If the cognitive workload is too high, a solution is delegation (Miller, 2014). Without trust, the user is not going to delegate, no matter how capable the robot is, under-trust may cause a poor use of the system (Parasuraman and Riley, 1997; Lee and See, 2004). Transparency is needed for understanding and trust, and trust is necessary for delegation (Kruijff et al., 2014; Ososky et al., 2014; Yang et al., 2017).

The cognitive workload reduction not always means a task performance improvement because of the automation-induced complacency (Wright et al., 2017). Complacency means over-trusting the system, and it is defined in Parasuraman et al. (1993) as “the operator failing to detect a failure in the automated control of system monitoring task.” For high levels of autonomy, transparency of the robot intent and reasoning is especially necessary to make the most of human-in-the-loop approaches, reducing complacency (Wright et al., 2017). Transparency and trust calibration can be improved by training (Nikolaidis et al., 2015) and a good interface design (Lyons, 2013; Kruijff et al., 2014). Some efforts to integrate trust into computational models can be found in Desai (2012) and Chen et al. (2018).

## 3. Transparency as observability and predictability of the system behavior

One of the most common ways of understanding transparency in human-robot interaction in shared autonomy framework is as observability and predictability of the system behavior: the understanding of what the system is doing, why, and what it will do next (Endsley, 2017).

What kind of information should be communicated in order to have a good level of transparency? The robot's state and capabilities must be communicated transparently to the human operator: what the robot is doing and why, what it is going to do next, when and why the robot fails when performing specific actions, and how to correct errors are essential aspects to be considered. In Kruijff et al. (2014) and Hellström and Bensch (2018), the authors go even further: their research explores, based on experimental data, not only what to communicate, but also communication patterns—how to communicate—for improving user understanding in a given situation.

Autonomy increases uncertainty and unpredictability about the system's state, and some authors understand transparency in the sense of predictability: “Transparency is essentially the opposite of unpredictability” (Miller, 2014) and “Transparency is the possibility to anticipate imminent actions by the autonomous system based on previous experience and current interaction” (Iden, 2017).

Other definitions, found in the literature, in the sense of observability are: “Transparency is the term used to describe the extent to which the robot's ability, intent, and situational constraints are understood by users” (Wortham et al., 2017), “Transparency is a mechanism to expose the decision-making of a robot” (Theodorou et al., 2016, 2017), and “the ability for the automation to be inspectable or viewable in the sense that its mechanisms and rationale can be readily known” (Miller, 2018).

Legible motion is “the motion that communicates its intents to a human observer” (Dragan et al., 2013), also referred as readable motion (Takayama et al., 2011), or anticipatory motion (Gielniak and Thomaz, 2011). Algorithmic approaches for establishing transparency in the sense of legibility can be found in Dragan et al. (2013, 2015a,b); Nikolaidis et al. (2016), and in Busch et al. (2017) based on optimization and learning techniques, respectively.

### 3.1. Robot-to-human transparency and robot-of-human transparency

Transparency about the robot's state information may be referred to as robot-to-human transparency (Lyons, 2013). One of the principles of user-centered design is to keep the user aware of the state of the system (Endsley, 2012; Villani et al., 2018). Robot-to-human transparency enables user-centered design. This mini-review is focused on this type of transparency.

There is also a robot-of-human transparency (Lyons, 2013), which focuses on the awareness and understanding of information related to humans. This concept of monitoring human performance is of growing interest to provide assistance, e.g., in driving and aviation. The term robot-of-human transparency is not widely used in literature. However, examples of robot-of-human transparency, without using the term directly, can be found in Lorenz et al. (2014); Lorenz (2015); Tsiourti and Weiss (2014); Dragan (2017); Wang et al. (2017); Doellinger et al. (2018); Goldhoorn et al. (2018); Gui et al. (2018), and Javdani et al. (2018). In Casalino et al. (2018) and Chang et al. (2018) a feedback of the intent recognition is communicated to the operator.

In Lyons (2013) and Lyons and Havig (2014) transparency is defined as a “method to establish shared intent and shared awareness between a human and a machine.” Since the main method to establish shared situation awareness and shared intent is the interface design, the next section is dedicated to the study of transparency as a property of the interface.

## 4. Transparency as a property of the interface

The Human-Automation System Oversight (HASO) model (Endsley, 2017) summarizes the main aspects, and its relationships, of Human-Automation Interaction (HAI). The place of transparency in this model is as a property of the interface. This model uses the three level situation awareness model (Endsley, 1995).

In Chen et al. (2014) Transparency is defined as an attribute of the human-robot interface “the descriptive quality of an interface about its abilities to afford an operator's comprehension about an intelligent agent's intent, performance, plans, and reasoning process.” The Situation Awareness Transparency (SAT) model (Chen et al., 2014), is based on Endsley (1995), and proposes three levels of Transparency:

- Level 1. Transparency to support perception of the current state, goals, planning, and progress.
- Level 2. Transparency to support comprehension of the reasoning behind the robot's behavior and limitations.
- Level 3. Transparency to support projection, predictions and probabilities of failure/success based on the history of performance.

Errors in the perception because the information was not clearly provided (lack of level 1 transparency) are the cause of a great amount of the situation awareness problems, which are the cause of failures due to human errors (Jones and Endsley, 1996; Murphy, 2014). The design of more transparent interfaces might improve situation awareness, reducing human errors.

Information to support transparency can be exchanged through different communication channels (Goodrich and Schultz, 2007): visual interfaces (Baraka et al., 2016; Walker et al., 2018), human-like explanation interfaces (the next sections is dedicated to explanation interfaces), physical interaction and haptics based interfaces (Okamura, 2018) (studied in the mechanical transparency section), or a combination in multimodal interfaces (Perzanowski et al., 2001; Oviatt et al., 2017). Lakhmani et al. (2016) study the possibility to add information about roles and responsibilities in the division of tasks to the SAT model, using a multimodal interface.

## 5. Transparency as explainability

Transparency can be achieved by means of human-like natural language explanations. In Kim and Hinds (2006) the definition given for transparency is “Transparency is the robot offering explanations of its actions.” Mueller sees explanation as one of the main characteristics of transparency (Mueller, 2016; Wortham et al., 2016).

According to the report about explainable artificial intelligence by the Defense Advanced Research Projects Agency (DARPA, 2016), the explanation interface should be able, at least, to generate answers to the user's questions:

- Why did the system do that and not something else?
- When does the system succeed? and
- When does the system fail?
- When can the user trust the system?
- How can the user correct an error?

Verbalization has been used to convert sensor data into natural language, to describe a route (Perera et al., 2016; Rosenthal et al., 2016) when the user requests information in a dialog, to explain a policy (Hayes and Shah, 2017), or in Zhu et al. (2017) to describe what a humanoid is doing in the kitchen.

Trust in robots is essential for the acceptance and wide utilization of robot systems (Kuipers, 2018; Lewis et al., 2018). Explanations improve usability and let the users understand what is happening, building the users' trust and generating calibrated expectations about the system's capabilities (Westlund and Breazeal, 2016). If systems can explain their reasoning, they should be easily understood by their users, and humans are more likely to trust systems that they understand (Sanders et al., 2014; Sheh, 2017; Fischer et al., 2018; Lewis et al., 2018).

## 6. Mechanical transparency

Wearable robots like exoskeletons are coupled to the user, and the robot moves with the wearer cooperatively (Awad et al., 2017; Anaya et al., 2018; Bai et al., 2018; Fani et al., 2018). In this case, the design should be able to follow the human movements minimizing resistive forces felt by the human, i.e., the design should be mechanically transparent. For example, in rehabilitation, a robot applies a force to a patient, and then the patient finishes the movement (Robertson et al., 2007; Jarrassé et al., 2009; Zhang et al., 2016; Beckerle et al., 2017).

The system is transparent if the robot follows exactly the human movement, without applying forces to the human. Transparency might be improved by human motion prediction (Jarrasse et al., 2008) and training (van Dijk et al., 2013). Trust calibration is needed to avoid the risk of overtrust in the capabilities of the exoskeletons (Borenstein et al., 2018).

Bilateral teleoperation, also named telerobotics, should enable the user to interact with a remote environment as if they were interacting directly. To interact with the remote environment a slave robot is used. The slave is controlled by a human operator using a human-machine interface or master, and the signals from master to slave, and the feedback from slave to master, are transmitted through a communication channel (Ferre et al., 2007; Goodrich et al., 2013; Hertkorn, 2015; Fani et al., 2018; Okamura, 2018).

In a transparent system, the slave tracks exactly the master, and the operator has a realistic (transparent) perception of the remote environment: the technical system should not be felt by the human (Hirche and Buss, 2007). Transparency can be degraded if there are time delays in the communication channel between the user and the remote environment (Lawrence, 1993; Hirche and Buss, 2007; Farooq et al., 2016). More details about transparency modeling for telerobotics can be found in Raju et al. (1989); Lawrence (1993); Yokokohji and Yoshikawa (1994); Ferre et al. (2007), and Slawinski et al. (2012).

When using brain computer interfaces (BCIs) (Bi et al., 2013; Rupp et al., 2014; Arrichiello et al., 2017; Burget et al., 2017) as the input device to teleoperate a robotic manipulator, the difficulty in decoding neural activity introduces delays, noises, etc., and specific techniques to improve transparency are required, such as the ones proposed in Muelling et al. (2017).

## 7. Transparency and ethically aligned design

Another aspect of transparency is in the sense of traceability and verification (Winfield and Jirotka, 2017; Wortham et al., 2017). Winfield and Jirotka (2017) propose that robots should be equipped with an ethical black box, the equivalent of the black box used in aircrafts, to provide transparency about how and why a certain accident may have happened, helping to establish accountability. This transparency could help disruptive technologies gain public trust (Sciutti et al., 2018).

Ethics and Standards are interconnected, and both fit into the broader framework of Responsible Research and Innovation. There is an IEEE Global Initiative for Ethically Aligned Design for Artificial Intelligence and Autonomous Systems, with a work group dedicated to Transparency (Bryson and Winfield, 2017; Grinbaum et al., 2017). In this initiative, Transparency is defined as “the property which makes possible to discover how and why the system made a particular decision, or in the case of a robot, acted the way it did.” The standard describes levels of transparency for autonomous systems for different stakeholders: users, certification agencies, accident investigators, lawyers, and general public.

European Union's new General Data Protection Regulation and the Recommendations to the Commission on Civil Law Rules on Robotics are examples of the increasing importance of ethically aligned designs. The first one creates the right to receive explanations (Goodman and Flaxman, 2016), and the second one recommends maximum transparency, predictability, and traceability (Boden et al., 2017; European Parlament, 2017).

## 8. Discussion

Marvin Minsky used the term “suitcase word” (Minsky, 2006) to refer to words with several meanings packed into them. Transparency is a kind of suitcase-like word, so we propose a categorization of the different meanings of transparency in shared autonomy identified in the state of the art. This categorization can be found in Table 1.

It can be observed that algorithmic approaches to establish and improve transparency are well developed, mature, and numerous in mechanical transparency and haptic interfaces. On the other hand, algorithms to establish transparency in the sense of observability, predictability, legibility, or explainability, or for other types of interfaces like brain computer interfaces, are not so numerous and have only been recently developed. Table 2 clusters a relevant selection of these algorithmic approaches.

**Table 2:** Main algorithmic approaches.

**Transparency in shared autonomy**	
**3. Transparency as observability and predictability of system behavior**	
Dragan et al., 2013, 2015a,b; Nikolaidis et al., 2016	Generating legible motion optimizing trajectories space
Busch et al., 2017; Buehler and Weisswange, 2018	Legibility using model-free methods
Takayama et al., 2011	Readibility based on handcoded animations
Cha et al., 2017; Kim and Fong, 2017; Ganesan et al., 2018	Visual cues and light signaling
**Robot-of-Human Transparency**	
Bethel and Murphy, 2008; Dragan, 2017; Roncone et al., 2017; Chen et al., 2018; Gui et al., 2018	Intent recognition
Gielniak and Thomaz, 2011; Lorenz et al., 2014; Bai et al., 2015; Matthews et al., 2017; Nikolaidis et al., 2017; Wang et al., 2017; Doellinger et al., 2018; Goldhoorn et al., 2018	Mutual adaptation
Breazeal et al., 2005; Li and Zhang, 2017; Li et al., 2017; Cha et al., 2018; Gildert et al., 2018; Haji Fathaliyan et al., 2018; Lakomkin et al., 2018	Implicit communication
**5. Transparency as explainability**	
Caminada et al., 2014; Rosenthal et al., 2016; Wortham and Rogers, 2017; Kuhner et al., 2018; Nikolaidis et al., 2018	Route/planning/navigation verbalization
MacMahon et al., 2006; Kollar et al., 2010; Matuszek et al., 2010; Duvallet et al., 2013, 2016; OSSwald et al., 2014; Hemachandra et al., 2015; Daniele et al., 2017; Suddrey et al., 2017; Nikolaidis et al., 2018; Sinha et al., 2018	Natural language grounding
**6. Mechanical transparency**	
**Wearables, Exoskeletons**	
Robertson et al., 2007; Jarrasse et al., 2008; Jarrassé et al., 2009; van Dijk et al., 2013; Kim and Rosen, 2015; Boaventura and Buchli, 2016; Zhang et al., 2016; Awad et al., 2017; Beckerle et al., 2017; Boaventura et al., 2017; Chen et al., 2017; Fong et al., 2017; Bai et al., 2018; Fani et al., 2018	Force feedback control with feedforward loop fed with predictive information, impedance and admittance controllers, electromyography methods
**Telerobotics**	
Raju et al., 1989; Lawrence, 1993; Yokokohji and Yoshikawa, 1994; Baier and Schmidt, 2004; Lee and Li, 2005; Hokayem and Spong, 2006; Monfaredi et al., 2006; Ferre et al., 2007; Goethals et al., 2007; Hirche and Buss, 2007; Polushin et al., 2007; Kim et al., 2010, 2013; Yalcin and Ohnishi, 2010; Baser and Konukseven, 2012; Franken et al., 2012; Na and Vu, 2012; Slawinski et al., 2012; Aracil et al., 2013; Baser et al., 2013; Goodrich et al., 2013; Meli et al., 2014; Pacchierotti et al., 2014; Hertkorn, 2015; Farooq et al., 2016; Park et al., 2016; Sun et al., 2016; Xu et al., 2016; Gopinath et al., 2017; Lu et al., 2017	Realistic perception of the remote environment through:adaptive impedance force control, stiffness observers, position-force controllers, four channel control, impedance reflection algorithms, coupled impedance controllers, cutaneous tactile force feedback
Burget et al., 2017; Muelling et al., 2017; Zhao et al., 2017	BCI, AR

Considering the challenges of transparency, several areas might be promising for future developments. The challenges of transparency in shared autonomy are different for high levels of autonomy and for low levels of autonomy.

For low levels of autonomy, the operator is doing almost everything directly, so the uncertainty and predictability are low, and transparency may be high, but there is a problem because the human cognitive workload to be aware of everything might become too high. The solutions might be:

- The use of intermediate levels of autonomy, so that the user might delegate some tasks (Miller, 2014). Trust is necessary for delegation, without trust, the user is not going to delegate, no matter how capable the robot is (Kruijff et al., 2014). Transparency helps build trust (Ososky et al., 2014).
- Improve the interfaces design to allow users to manage the information available, to obtain a high level of understanding of what is going on.
- Learn from the experience. If a robot requests human support in a difficult situation, the human actions could be stored and executed the next time the robot faces the same situation.

For high levels of autonomy, human is delegating almost everything, so the uncertainty and predictability are high, and the transparency may be low. The operator cognitive engagement and attention might become low (Endsley, 2012; Hancock, 2017), and it might cause problems detecting failures (complacency effect) (Parasuraman et al., 1993), and recovering manual control from automation failure (Lumberjack effect)(Onnasch et al., 2014; Endsley, 2017). The solutions might be:

- The use of intermediate levels of autonomy.
- Increase of transparency of the system's intent and reasoning, including information beyond the three levels SAT model.
- Increase robot-of-human transparency to recognize human attention reduction.
- Training to avoid the out-of-the-loop performance problem, and calibrate the trust in the system.

## 9. Conclusions

The current research about transparency in the shared autonomy framework has been reviewed, to provide a general and complete overview. The next ways of understanding transparency in human-robot interaction in the shared autonomy framework have been identified in the state of the art:

- Transparency as the observability of the system behavior, and as the opposite of unpredictability of the state of the system. The human understanding of what the system is doing, why, and what it will do next.
- Transparency as a method to achieve shared situation awareness and shared intent between the human and the system. The main methods to improve shared situation awareness are interface design and training.
- Robot-to-human transparency (understanding of system behavior) vs. robot-of-human transparency (understanding of human behavior). This work has focused on the first one.
- Transparency as a property of the human-robot interface and the transparency situation awareness model. Transparent interfaces can be achieved through natural language explanations.
- Mechanical transparency used in haptics, bilateral teleoperation, and wearable robots like exoskeletons.
- Transparency as traceability and verification.

The benefits of transparency are multiple: transparency improves system performance and might reduce human errors, builds trust in the system, and transparent design principles are aligned with user-centered design, and ethically aligned design.

## Author contributions

VA and PdP contributed conception and design of the study. VA and PdP contributed to manuscript preparation, revision and approved the submitted version. VA is responsible for ensuring that the submission adheres to journal requirements, and will be available post-publication to respond to any queries or critiques.

### Conflict of interest statement

The authors declare that the research was conducted in the absence of any commercial or financial relationships that could be construed as a potential conflict of interest.
